# The Evolution of Central Retinal and Choroidal Thickness in Acute Anterior Uveitic Patients with Spondyloarthropathy

**DOI:** 10.1155/2018/9136017

**Published:** 2018-08-06

**Authors:** Zsuzsanna Szepessy, Árpád Barsi, Kinga Kránitz, Zoltán Zsolt Nagy

**Affiliations:** ^1^Department of Ophthalmology, Semmelweis University, Budapest, Hungary; ^2^Department of Photogrammetry and Geoinformatics, Budapest University of Technology and Economics, Budapest, Hungary

## Abstract

**Purpose:**

To describe and correlate the degree of anterior segment inflammation with central retinal and choroidal thickness throughout the treatment period (in the course of follow-up) in the eyes affected with acute anterior uveitis in the patients with seronegative spondyloarthropathy (subgroup: ankylosing spondylitis).

**Methods:**

Thirty eyes of 30 consecutive Caucasian patients with HLA-B27-associated acute anterior uveitis were included in this study. The flare, AC cell number, and central retinal/choroidal thickness were determined at each visit by optical coherence tomography and laser flare photometry. Treatment consisted of topical corticosteroids. Statistical analysis was performed by MathWorks Matlab software.

**Results:**

In the follow-up period, central retinal thickness was increased in the first 9-10 days and then decreased until stabilization (after 5-6 weeks). The flare and AC cell number decreased rapidly at the beginning of the treatment, in the first 10 days, and thereafter, slower decrease could be observed until complete resolution of inflammation. Statistically significant, positive correlation was found between initial laser flare value and maximal central retinal thickness (*r*=0.881, *p* < 0.001). Positive correlation between flare and retinal thickening was observable throughout the treatment period. Central choroidal thickness was decreased also significantly during the follow-up (*p* < 0.001).

**Conclusions:**

The retina and choroid may play a biomarker function in the anterior segment inflammation of the eye in the patients with seronegative spondyloarthropathy.

## 1. Introduction

Spondyloarthropathy (SpA) is a group of chronic inflammatory rheumatic diseases, which are characterized by common clinical symptoms and genetic similarities [1]. Spondyloarthropathy is genetically linked (90% of cases), and the strongest contributing factor is the HLA B27 antigen [1, 2]. Ocular inflammation is a common extra-articular manifestation of seronegative spondyloarthropathy. Anterior uveitis is one of the most important features with a prevalence of 25–30% in the SpA patients [1, 2]. Five major subtypes of SpA are recognized on the basis of recently proposed classification criteria (European Spondyloarthropathy Study Group). In this study, one of the most common subtypes of SpA ankylosing spondylitis (AS) was studied intentionally.

Acute inflammatory process in the anterior segment, as opposed to chronic anterior uveitis, is rarely associated with significant macular edema, but subclinical retinal thickening has already been described [3, 4]. Our earlier results showed also close linear correlation between central foveal thickness and anterior segment inflammation (laser flare photometry values) at the beginning of the first anterior uveitic attack in the patients with HLA-B27-related spondyloarthropathy [3].

In recent years, some studies have used EDI-OCT to evaluate choroidal changes in ocular inflammatory disorders [5, 6]. Some authors have reported that the patients with active Vogt–Koyanagi–Harada disease [5] and Behçet disease [6] exhibit a markedly thickened choroid. Currently, there are a limited number of reports on choroidal thickness change in anterior uveitis [7].

The aim of this study was to describe the evolution of retinal and choroidal thickness in eyes affected with acute anterior uveitis in the course of follow-up in the patients with seronegative spondyloarthropathy. Our goal was to evaluate the correlation between flare and retinal/choroidal thickening throughout the treatment period.

## 2. Materials and Methods

This prospective study was performed at the Department of Ophthalmology of the Semmelweis University (Budapest, Hungary) on 30 eyes of 30 consecutive Caucasian patients (24 males and 6 females; age: 27–50 years; mean age: 35 years) with acute anterior uveitis for the first time.

Before their first uveitic attack, rheumatic diseases, seronegative spondyloarthropathy (subtype: ankylosing spondylitis), have been diagnosed by rheumatologist. All patients were HLA-B27 positive. The patients with a history of psoriatic arthritis (PsA), reactive arthritis (ReA), arthritis associated with inflammatory bowel disease (AIBD), other systemic diseases, intermediate uveitis, posterior uveitis, intraocular surgery, macular disease (e.g., epiretinal fibrosis and diabetic maculopathy), and amblyopic eyes were excluded from the study. Ankylosing Spondylitis Disease Activity Score (ASDAS) of the patients in this study was less than 2.0 to define “moderate disease activity.” All of them were treated with systemic nonsteroidal anti-inflammatory drugs by rheumatologist.

All participants were treated in accordance with the tenets of the Declaration of Helsinki. Written informed consent was obtained from all participants in this study.

All patients underwent a comprehensive eye examination, including best corrected visual acuity (BCVA), measurement of the intraocular pressure, and slit-lamp and indirect fundus examination after pupil dilation. The axial length (AL) of the eyes was measured using optical biometry (Lenstar LS 900 Haag-Streit AG, Switzerland).

**Figure 1:**
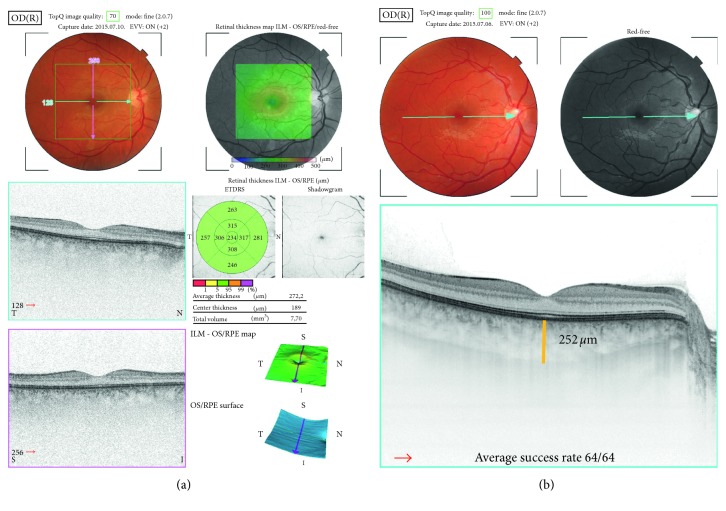
Retinal (a) and choroidal (b) thickness measurements by swept-source optical coherence tomograph (DRI OCT Triton, Topcon Co., Tokyo, Japan).

OCT examinations were performed on each eye using EDI-OCT (Optovue Inc., Fremont, CA, USA) and swept-source optical coherence tomograph (DRI OCT Triton, Topcon Co., Tokyo, Japan) by the same operator through dilated pupils at least 5 mm in diameter. The retinal and choroidal thickness measurements for the central ETDRS region of the macula were recorded at each visit throughout the treatment period (Figure 1).

Quantitative measurements of anterior segment aqueous flare and inflammatory cells were conducted using a laser flare meter (Kowa FC-600). Flare values and cell counts were measured at 30 minutes after pupillary dilation. The same examiner has obtained five measurements from each eye, and the results were averaged after excluding all measurements with artefacts.

SUN (Standardization of Uveitis Nomenclature) Working Group standardized the grading of anterior segment cells and flare. For anterior segment cells, in a field size of 1 × 1 mm slit beam, the following grades were described: 0 (<1 cell), 0.5+ (1–5 cells), 1+ (6–15 cells), 2+ (16–25 cells), 3+ (26–50 cells), and 4+ (>50 cells). The number of the anterior segment cells was also determined, counted using SUN criteria.

All uveitic eyes were initially managed with topical corticosteroid drops (prednisolone acetate 1%) every 1–6 hours depending on severity and cyclopentolate (5 mg/ml) three times a day. Steroid was withdrawn slowly, and corticosteroid drops were continued until the cellular reaction is absent. Cycloplegics were discontinued when the cellular reaction is subsiding. The patients were followed daily until manifest response to local treatment was observed (defined as a decrease of anterior segment cells on slit-lamp examination or a decrease of more than 20% from the baseline in laser flare photometry) and weekly thereafter until complete resolution of inflammatory activity.

Statistical analysis was performed by MathWorks Matlab 2012b software and its Statistics Toolbox. A *p* value of less than 0.05 was considered as statistically significant.

## 3. Results

The clinical signs data at the beginning of the first inflammatory episode (best corrected visual acuity (BCVA), flare, anterior segment cells (AC), intraocular pressure, central retinal thickness, and central choroidal thickness) are shown in Table 1.

As expected, flare and anterior segment cell number are significantly lower between initial flare/AC cell number and final flare/AC cell number (*p* < 0.001).

**Figure 2:**
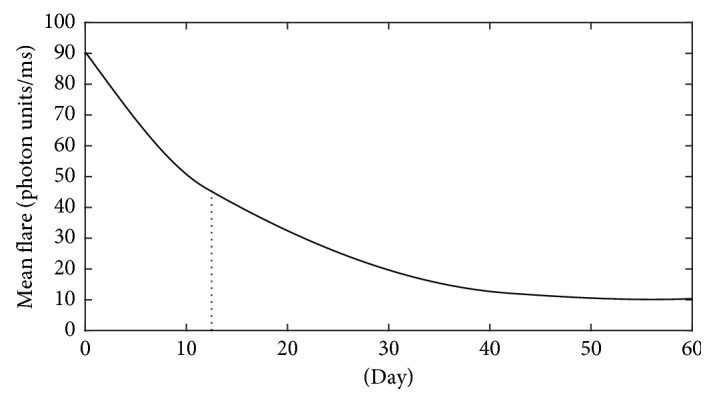
The evolution of the mean laser flare value in the course of follow-up of the acute anterior uveitic patient with ankylosing spondylitis. The mean initial laser flare value is reduced by 50% after 12 days.

**Figure 3:**
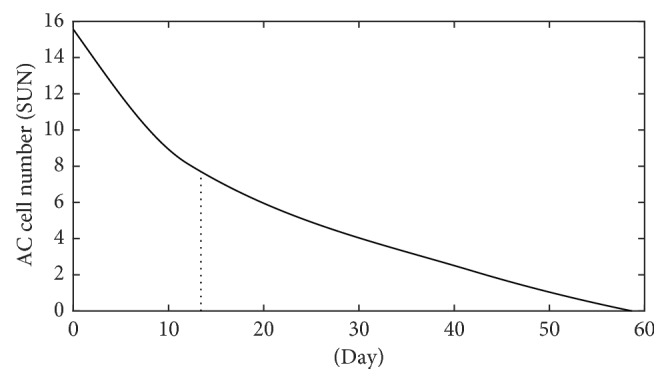
The evolution of AC cell number (SUN) during the treatment period of the acute anterior uveitic patient with ankylosing spondylitis.

Figure 2 presents the evolution of the mean laser flare value in the course of follow-up. AC cell number in the anterior uveitic eyes is significantly decreased during the treatment period (*p* < 0.001), as shown in Figure 3. The mean initial laser flare value is reduced by 50% after 12 days. Flare and AC cell number are decreased rapidly at the beginning of the treatment, in the first 10 days, and thereafter decreased slowly until complete resolution of inflammation.

**Figure 4:**
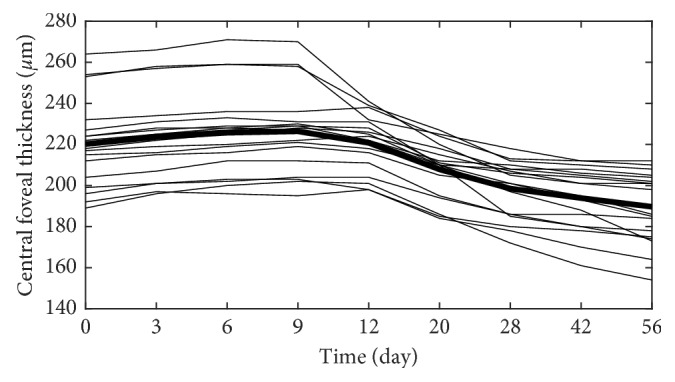
The evolution of mean central retinal thickness in the treatment period of the acute anterior uveitic patient with ankylosing spondylitis (the mean central retinal thickness value is represented by a bold line).

The central retinal thickness was significantly decreased (*p* < 0.001) between the beginning and the end of the follow-up period.  Figure 4 shows the evolution of mean central retinal thickness in the treatment period. In the follow-up period, central retinal thickness increased a little in the first 9-10 days, followed by a progressive decrease until stabilization (after 5-6 weeks). The mean difference between the initial and maximal retinal thickness was 8.367 *µ*m.

**Figure 5:**
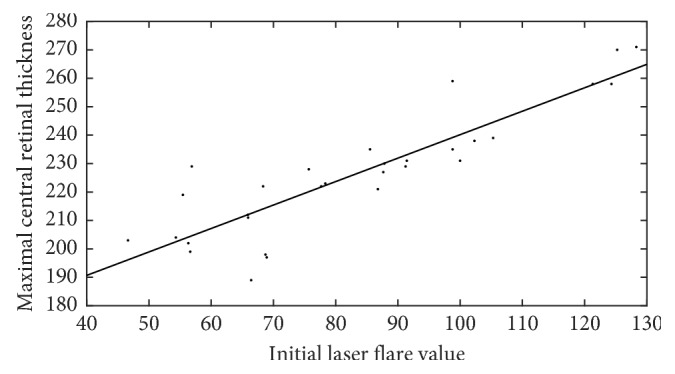
Statistically significant, positive correlation was found between initial laser flare value and maximal central retinal thickness (*r*=0.881, *p* < 0.001) in the acute anterior uveitic eyes.

**Figure 6:**
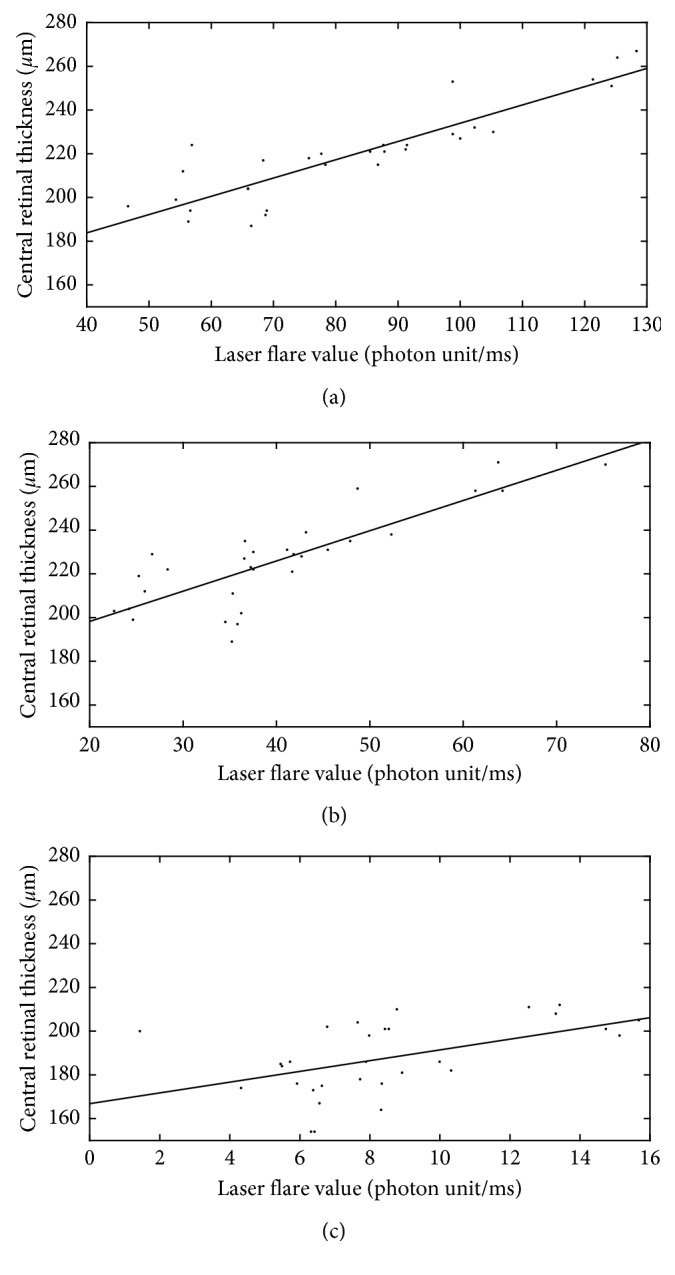
Positive linear correlation between flare and retinal thickness remained throughout the treatment period: (a) 0 days; (b) 10 days; (c) 30 days.

Statistically significant, positive correlation was found between initial laser flare value and maximal central retinal thickness (*r*=0.881, *p* < 0.001) in this homogenous group of uveitic eyes, as shown in Figure 5.  Figure 6 demonstrates that the positive correlation between flare and retinal thickness remained throughout the treatment period.

**Figure 7:**
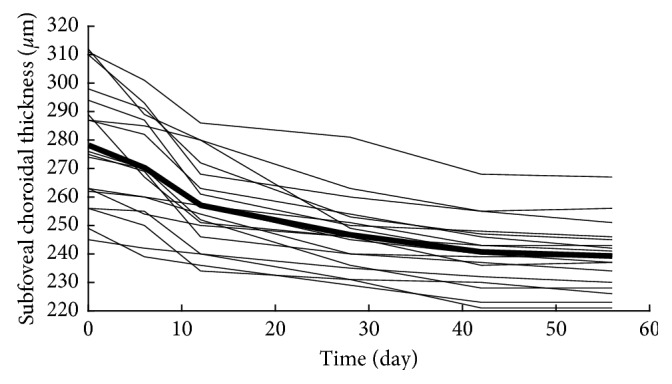
The evolution of mean central (subfoveal) choroidal thickness in the treatment period of the acute anterior uveitic patient with ankylosing spondylitis. The mean central choroidal thickness value is represented by a bold line.

**Figure 8:**
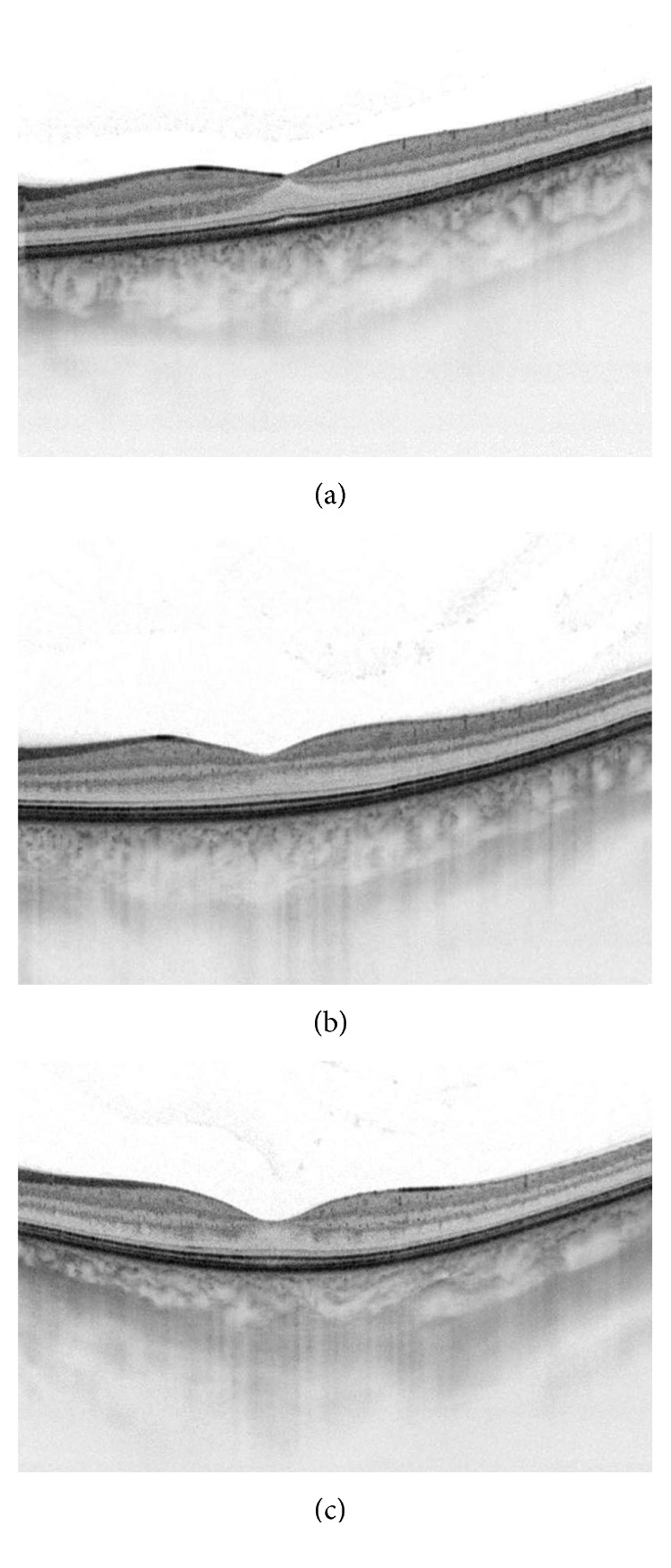
Swept-source OCT cross-sectional images of a 35-year-old male anterior uveitic patient with seronegative spondyloarthropathy: (a) at the beginning of the anterior uveitis (0 days, central retinal thickness: 234 *µ*m, central choroidal thickness: 298 *µ*m); (b) after 10 days in the treatment period (10 days, central retinal thickness: 248 *µ*m, central choroidal thickness: 267 *µ*m); (c) after a month (30 days, central retinal thickness: 219 *µ*m, central choroidal thickness: 242 *µ*m).

Finally, the central (subfoveal) choroidal thickness was significantly decreased (*p* < 0.001) in the treatment period, as shown in Figures 7 and 8.

## 4. Discussion

Our study demonstrated a pattern of evolution of the retinal and choroidal thickness and flare values throughout the treatment period in the eyes affected by HLA-B27-associated acute anterior uveitis (AAU) (subgroup: ankylosing spondylitis).

The most common posterior segment findings in AAU are subclinical increase in retinal thickness, choroidal thickness, and macular edema. A number of studies have investigated retinal thickness of the anterior uveitic patients at the onset of the inflammation [3, 4, 8–10]. For instance, Moreno-Arrones et al. [4], Balaskas et al. [8], and our earlier study [3] also described statistically significant difference in retinal thickness between the uveitic eyes and the fellow eyes for all OCT subfields. In contrast, de Lahitte et al. described only perifoveolar, pericentral thickening in the patients with juvenile idiopathic arthritis-associated chronic anterior uveitis [11].

Our earlier results have shown that changes of the anterior segment inflammation might cause subclinical retinal changes [3]. We suppose that ocular inflammation may produce a breakdown of the blood-retinal barrier and increase vascular permeability which generates retinal thickening.

In our current study, the evolution of the mean central foveal thickness and anterior segment flare values in the treatment period was shown. In this follow-up period, central retinal thickness increased a little in the first 10 days, followed by a progressive decrease until stabilization (after 5-6 weeks). The mean difference between the initial and maximal retinal thickness was 8.367 *µ*m in our study. The maximal retinal thickness of the affected eyes was observed on day 9-10 from the beginning of the inflammation.

This time lag between the retinal thickening and the onset of inflammation may be the explanation of the development of Irvine–Gass syndrome, whereas macular edema presents several weeks following cataract surgery (inflammatory agents). The other typical phenomenon is diabetic macular edema, which develops slowly as an accumulative response of the retina for multiple factors, that is, inflammation. The main factor triggering macular edema is the release of inflammatory mediators. In chronic uveitis, the repeated/long-lasting inflammatory stimuli could result in additive effects on the retinal thickening. In alignment, Balaskas et al. presented also an increase in retinal thickness over a period of 17 to 25 days in the AAU patients [8]. An important result is that retinal thickness, as measured by OCT, is a useful clinical parameter to monitor response to treatment. Changes in retinal thickness in the AAU patients demonstrate the characteristic pattern of the retinal response to inflammation and therapy. The reply of the retina for the inflammatory agents seems to be delayed.

Anterior segment cells were counted using SUN criteria, but we have used also laser flare photometry (LFP) to eliminate the subjectivity in measurements of anterior segment cells by slit-lamp biomicroscopy [12, 13]. LFP results were always interpreted in conjunction with the clinical observations. Flare and AC cell number were decreased rapidly at the beginning of the treatment, in the first 10 days, and thereafter, slower decrease could be observed until complete resolution of inflammation. While the inflammation values decreased rapidly in the first 10 days, the retinal thickness increased slightly in this period; however, after 10 days, both parameters decreased during the follow-up period.

The mean initial laser flare value was reduced by 50% after 12 days in our study. In the literature, Balaskas et al. [8] described a 50% reduction of flare after 14 days, and de Ancos et al. [14] observed the same drop after 2 days. Contradictory results may come from a different initial severity of the anterior segment inflammation in the studies. According to our results, retinal thickness increased slightly in the first 10 days when flare reduction was remarkable; however, significant changes in retinal thickness values could be observed later in the treatment period.

As shown in our earlier study, positive correlation was found between initial central foveal thickness and initial laser flare values of the uveitic patient with HLA-B27-related spondyloarthropathy [3]. Gonzales et al. also described that flare was significantly higher in the uveitic patients with macular edema than in patients without macular edema [12].

In this study, positive correlation was demonstrated between flare and central retinal thickness throughout the treatment period, and also, close correlation was shown between initial laser flare values and maximal central retinal thickness. Accordingly, the retina may be an indicator not only of posterior segment inflammation activity but also the anterior segment inflammation.

This study also showed a significant decrease in choroidal thickness in the eyes with active anterior uveitis following anti-inflammatory treatment. It is widely known that the choroid is influenced by ocular inflammatory conditions. Several authors have suggested increased vascular permeability in the posterior segment and choroidal effusion as the mechanisms of choroidal thickening during ocular inflammation [15, 16]. In the present study, the mean values of subfoveal choroidal thickness in the eyes with AAU changed significantly from 278.166 to 239.222 *µ*m. This study showed a significant decrease in choroidal thickness in the eyes with active anterior uveitis after treatment.

Our results indicate that subclinical choroidal inflammation may also be present in active anterior uveitis. These findings suggest that choroidal evaluation may be useful for monitoring disease activity.

Nevertheless, a larger case series could contribute to a more sophisticated statistical evaluation of the patients with AAU, but their common inflammatory pattern enables us to study the inflammation effect of the retina and the choroid.

In conclusion, swept-source OCT is also a useful tool in monitoring subclinical retinal and choroidal thickening in anterior uveitis with the SpA patients in the treatment period. It may indicate that the retina and choroid play a biomarker function in anterior segment inflammation but the reply of the retina for the inflammatory agents seems to be delayed.

## Figures and Tables

**Table 1:** Clinical signs of the first anterior uveitic attack in ankylosing spondylitis patients.

	BCVA (Snellen)	Flare (photon/unit)	AC cell number (SUN)	IOP (Hgmm)	Central retinal thickness (*µ*m)	Central choroidal thickness (*µ*m)
Minimum	0.6	66.63	12	11	189.00	245.00
Maximum	1.0	135.23	35	18	262.00	312.00
Mean	0.90	90.52	15.56	14.24	220.171	278.166
Standard deviation	0.120	25.315	7.695	2.450	21.056	21.475
Median	0.9	90.23	16	14	219.3	275.5

## Data Availability

The data used to support the findings of this study are included within the article.
